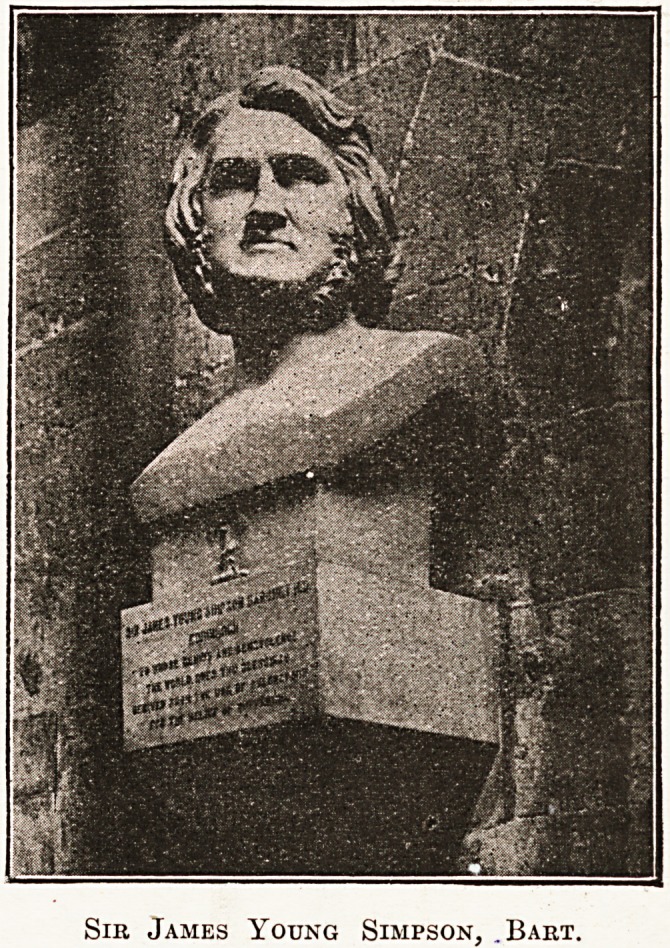# Memorials to Medical Men: Westminster Abbey

**Published:** 1915-07-31

**Authors:** 


### MEMORIALS TO MEDICAL MEN.

### I.—Westminster Abbey.

It is curious that the monuments and memorials to doctors are among the latest which have found a place in Westminster Abbey. Perhaps the reason may be found in Sir Godfrey Kneller's dying words, "By God, I will not be buried in Westminster . . . they do bury fools there"; but the fact remains that it was not until the eighteenth century that their memorials began to appear upon the walls of the Abbey. Some earlier traces of diseases which our ancestors were powerless to combat may, however, be found recorded at Westminster. In the Cloisters an enormous blue gravestone, familiarly known as "Long Meg," covers the remains of Abbot Bircheston and twenty-six monks who fell victims to the Black Death which ravaged England in 1348-9. In the seventeenth century the effects of the dreaded smallpox were

Sir Peter Warren.

even more widespread, and a quaint epitaph in the Little Cloisters commemorates "Mr. Thomas Smith, Bachelor of Arts," who "through ye spotted veil of the smallpox rendered a pure and unspotted soul to God," while the bust of Admiral Sir Peter Warren (d. 1732) is realistically pitted with the marks of the disease.

First Doctor to be Buried in the Abbey.

The first doctor of importance who was buried in the Abbey was Thomas Willis (d. 1675), one of the original founders of the Royal Society, whose work on the anatomy of the brain, a portion of which is still known as the "circle of Willis," was the most comprehensive treatise on the subject then published. It had the further distinction of having been illustrated by Sir Christopher Wren. Not far from his grave in the nave is a monument and bust to one of the best known physicians of his day, Dr. Richard Mead (1673-1754), a man who, as Dr. Johnson once remarked, "lived more

Richard Mead.

in the broad sunshine of life than almost any man." He was one of the earliest supporters of inocula-

Hugo Chamberlen.

tion for smallpox, and attended Sir Isaac Newton in his last illness. Horace Walpole, however, said that he "had nothing but pretensions," perhaps remembering how Mead had refused to attend his father, Sir Robert Walpole, until the latter had ordered the release from the Tower of the Jacobite Dr. John Freind, the author of the "History of Physic," who is also commemorated by a bust in the Abbey. It was not the only time that Mead defended his friend, for on another occasion he fought a duel with the eccentric Thomas Woodward (d. 1728), Professor of Physic at Gresham College, who had criticised the "History of Physic," and would have killed him had not the bystanders intervened. Woodward, who now lies buried within a few steps of his assailant, has other claims to remembrance, for he has been called the founder of English geology, and the Chair of Geology at Cambridge is named after him. A contemporary of these was Hugo Chamberlen (d. 1728), Court physician to the Stuarts, who has an imposing monument in the north aisle of the choir.

Four Scotch doctors have their memorial in the Abbey. William Buchan (1729-1805), the author of the popular "Domestic Medicine," has a tablet in the West Cloister; a bust by Chantrey in the Chapel of St. Andrew commemorates Mathew Baillie (1761-1823), the author of the "Morbid Anatomy," and brother of the poetess, Joanna Baillie, while Sir John Pringle (1707-1782), who did much to reform military medicine and sanitation, has a tablet in the south transept. A bust in St. Andrew's Chapel commemorates Sir James Young Simpson (1811-1870), to whom we owe the use of chloroform as an anæsthetic.

Sir John Pringle.

John Hunter.

There remains to be mentioned the greatest of the surgeons and doctors buried in the Abbey, John Hunter (1728-1793), the "founder of scientific surgery." A strange story is connected with his burial in the Abbey, for he was at first interred in the vaults of St. Martin-in-the-Fields. Nearly seventy years later, however, the churchwardens decided finally to close up the vaults, and gave public notice that anyone having relatives buried there might, if they so wished, remove their remains. Seeing this notice, the well-known naturalist, Frank Buckland, a son of a former Dean of Westminster, determined to try to rescue Hunter's remains, and have them reinterred in the Abbey. It proved a more difficult task than he had anticipated, and for sixteen days he worked unceasingly in the dismal and unhealthy vaults to find the coffin. By the light of a bull's-eye lantern he inspected no fewer than three thousand coffins before at last, when he had almost given up hope

Sir James Young Simpson, Bart.

and only five coffins remained, his light fell on the name of the great surgeon. On March 28, 1859, the Royal College of Surgeons re-interred the body of their illustrious founder in the nave of the Abbey, in a grave next to that of Ben Jonson.

Lister and the Abbey.

This poet and friend of Shakespeare, as is well known, was at his own request buried standing upright, and it is worth noting that in digging Hunter's grave Ben Jonson's skull was found with traces of red hair still upon it.

There can be no dispute that it was but fitting that the great work of Lord Lister, the second founder of modern surgery, should be commemorated in the Abbey, and although his personal wish prevailed and he found a resting-place elsewhere, a medallion from the hand of Sir Thomas Brock has been commissioned, and will shortly be placed among the memorials of the mighty dead in the Abbey church.

## Figures and Tables

**Figure f1:**
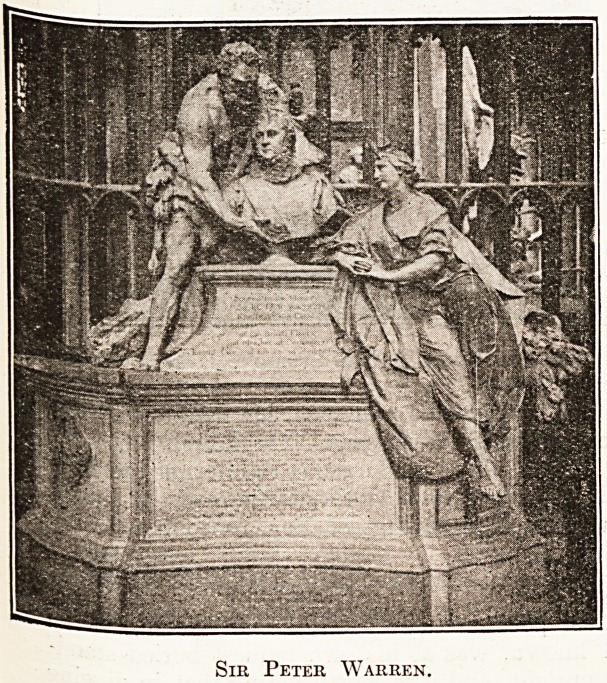


**Figure f2:**
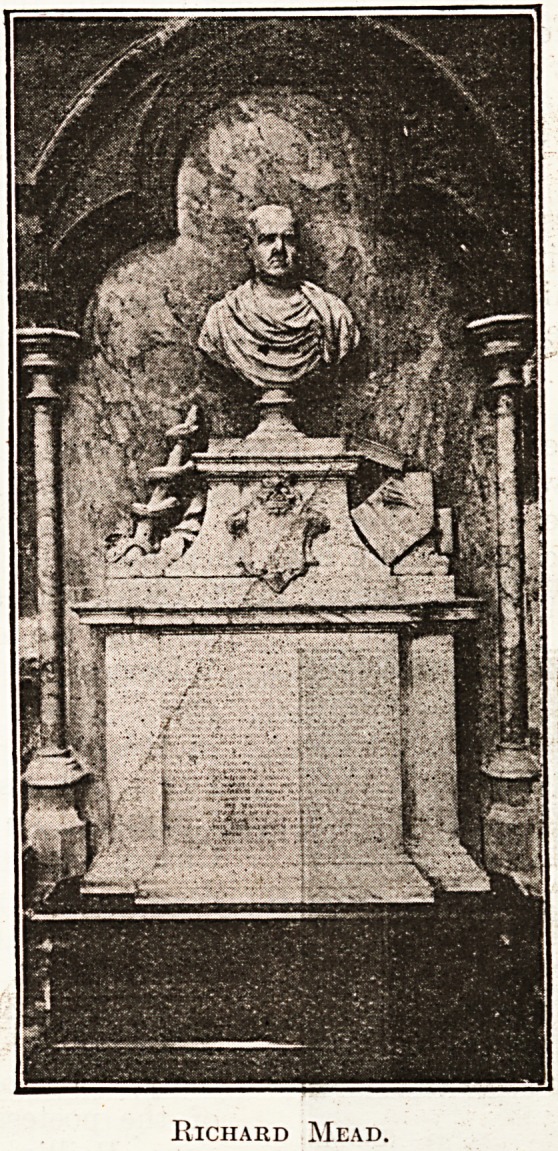


**Figure f3:**
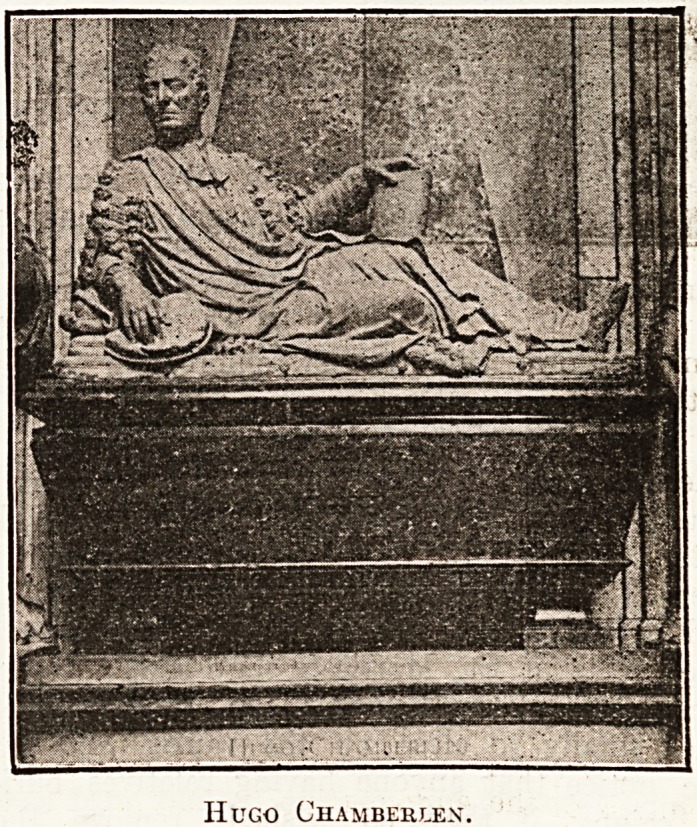


**Figure f4:**
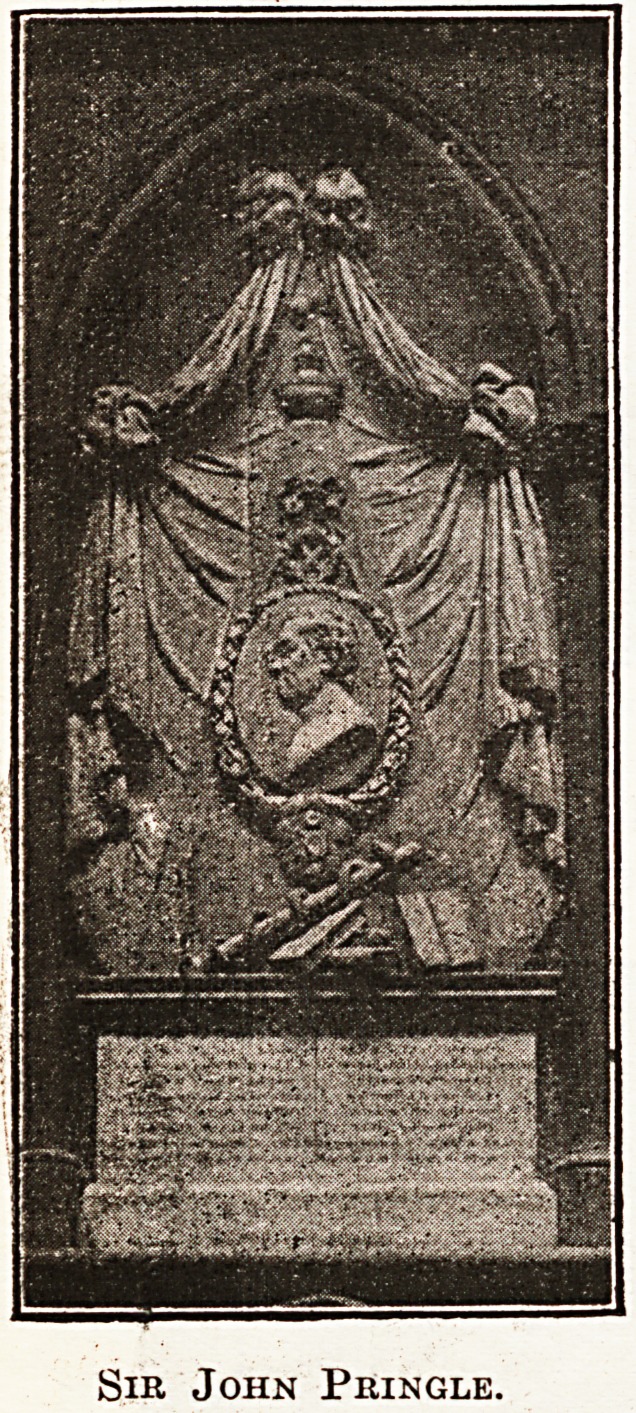


**Figure f5:**